# Effect of Strain Heterogeneities on Microstructure, Texture, Hardness, and H-Activation of High-Pressure Torsion Mg Consolidated from Different Powders

**DOI:** 10.3390/ma11081335

**Published:** 2018-08-01

**Authors:** Subrata Panda, Laszlo S. Toth, Jianxin Zou, Thierry Grosdidier

**Affiliations:** 1 Laboratoire d’Etude des Microstructures et de Mécanique des Matériaux (LEM3), Université de Lorraine, CNRS, Arts et Métiers ParisTech, LEM3, 57000 Metz, France; laszlo.toth@univ-lorraine.fr; 2 Laboratory of Excellence on Design of Alloy Metals for low-mAss Structures (DAMAS), Université de Lorraine, 57073 Metz, France; subrata.panda@univ-lorraine.fr; 3 National Engineering Research Center of Light Alloy Net Forming & State Key Laboratory of Metal Matrix Composites, School of Materials Science and Engineering, Shanghai Jiao Tong University, Shanghai 200240, China; zoujx@sjtu.edu.cn

**Keywords:** magnesium powders, HPT consolidation, microstructure, hardness, H-activation

## Abstract

Severe plastic deformation techniques, such as high-pressure torsion (HPT), have been increasingly applied on powder materials to consolidate bulk nanostructured materials. In this context, the aim of the present study is to compare the plastic deformation characteristics during HPT of two distinct Mg-based powder precursors: (i) atomized micro-sized powder and (ii) condensed and passivated nanopowder. Dynamic recrystallization could take place during HPT consolidation of the atomized powder particles while the oxide pinning of the grain boundaries restricted it for the condensed powder. Consequently, there have been substantial differences in the development of the microstructure, texture, local strain heterogeneities, and hardness in the two types of consolidated products. Different types of local strain heterogeneities were also revealed in the consolidated products. The associated diversity in microstructure within the same consolidated product has been demonstrated to have an effect on the hydrogen activation kinetics to form hydrides for these Mg-based materials that could be suitable for solid state H-storage applications.

## 1. Introduction

Severe plastic deformation (SPD) techniques are well known for their effective potential to produce ultrafine-grained/nanocrystalline bulk materials with enhanced mechanical and functional properties for a large variety of metal systems [1,2]. It is only recently that they have been employed for powder consolidation, not only because of their ability to fabricate bulk nanostructured materials but also for their capability of low-temperature solid-state densification under large shear strains [3,4,5]. Among the existing SPD processes, high-pressure torsion (HPT) [6] is an effective process for powder consolidation due to its unique working principle: simple shear deformation of individual particles by introducing extremely large amount of shear strains under high hydrostatic pressures. In addition, compared to other SPD routes, for example, equal-channel angular pressing (ECAP), in HPT it is simpler to control the processing parameters and is also easier to apply on powder materials. Therefore, a significant amount of research involving HPT assisted powder consolidation has been conducted on a wide range of material systems such as metallic materials [7,8,9,10], metal-matrix composites [11,12,13,14,15], etc.

Magnesium-based materials are regarded as potential structural elements in the automotive, aerospace, and biomedical applications mainly due to their lightweight, low cost and excellent specific strength [16]. The design and manufacture of high quality structural components made of Mg remain challenging. In general, Mg alloys are produced by casting processes, which generate coarse and segregated microstructures with residual porosities; factors that are limiting the mechanical strength [17,18]. Also, the significant grain growth occurring during high-temperature post-treatments may alter the materials properties. In contrast, the powder metallurgy (PM) processes represent an effective way for mechanical improvement through reduction in segregation scale, improvement in densification and microstructural refinement [19,20,21]. One of the major drawbacks of the PM process in Mg is the lack of ductility inherited from the presence of oxides at the surface of the powder particles which are difficult to remove during “conventional” sintering [20,22,23]. Therefore, an additional plastic deformation step is generally required [24,25]. Among the plastic deformation processes, solid-state sintering through SPD techniques are the most desirable, offering real alternatives to the conventional press/sinter routes. Indeed, the particle-particle interactions during severe plastic deformation are mainly governed by shearing rather than diffusion to achieve the required bonding and, thus, the bonding can be obtained at much lower temperatures.

To date, there have been limited studies [14,15,26,27,28,29] involving Mg based powder consolidation through SPD processing routes. Moss et al. [26] employed the ECAP technique to produce bulk materials by consolidating Mg and its alloy powders. Back pressure equal channel angular consolidation (BP-ECAC) of micrometer size Mg powder at 200 °C using route C produced a dense consolidated Mg sample with enhanced mechanical properties [27]. Also, it has been observed that ECAP processing of pure Mg and Mg-Ti powders led to fully dense consolidated samples with efficient structural refinement comparable to those obtained by mechanical milling [28]. An Mg_95_Zn_4.3_Y_0.7_ alloy was processed by HPT consolidation at room temperature as well as at 100 °C, and the superior mechanical properties of this alloy were associated with the ultrafine-grained microstructures accompanied with a high density of dislocations and also due to the significant equilibrium grain boundaries attained at elevated temperature [29].

Although the feasibility of HPT processing is, in the vast majority of cases, appreciated for fabricating thin samples with thickness of around 1 mm [5,7,8,9,11,12,13], some recent studies have revealed that there are significant structural heterogeneities across the disk-thickness when relatively thick samples are subjected to HPT [10,30,31,32]. In particular, Zhao et al. [10] reported the development of gradient microstructures in consolidated bcc-iron powder while using a constrained HPT facility. Substantial shear strain localization at the middle thickness of the HPT disks was noticed while processing bulk Mg sample through quasi-constrained HPT conditions [32].

In this context, motivated by the potential advantages of SPD powder consolidation, and also by the difficulties associated with the conventional processing of Mg-based materials, the present study focuses on HPT consolidation of two kinds of Mg powder precursors: atomized micro-sized powder produced by gas atomization and condensed ultrafine powder obtained by arc-plasma condensation. Since the initial powder particles were very different [33], it could be expected that the nature of the initial powder precursor influenced the powder consolidation and the associated developments in microstructures and mechanical properties in the resultant consolidated products. Following the pioneering works of Skripnyuk et al. using ECAP and high-energy ball milling (HEBM) [34,35], several routes of SPD processing have been employed for enhancing the hydrogen sorption properties of Mg and its alloys [33,36,37,38]. Here, the effect of HPT on the Mg powders and the associated structural heterogeneities on the kinetics of hydrogen activation was analyzed.

## 2. Materials and Methods

### 2.1. Materials and Consolidation by HPT

**Figure 1:**
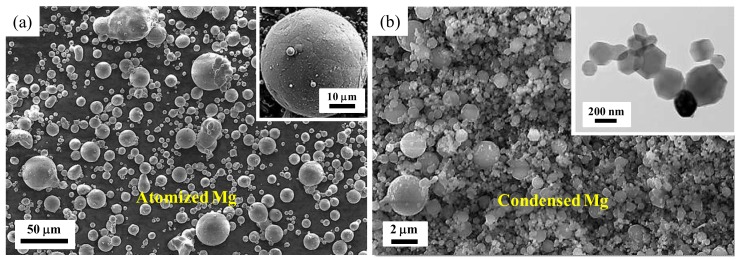
Morphological features of the initial powder precursors: (**a**) scanning electron microscopy (SEM) image of the atomized Mg particles accompanied with a magnified image of a particle showing grain structures (inset); (**b**) SEM image and a typical bright field tunneling electron microscopy (TEM) image (inset) of the condensed Mg particles.

Two kinds of Mg powder precursors—atomized micro-sized powder produced by gas atomization and condensed ultrafine powder obtained by arc-plasma condensation—were employed for HPT-assisted powder consolidation at room temperature. Commercial purity Mg (99.8%) was gas atomized by SFM SA (Martigny, Switzerland) to produce micro-sized Mg powder with a particle size distribution in the range of 10–70 µm. Figure 1a shows a scanning electron microscopy (SEM) image illustrating the morphological aspects of the micro-sized powder particles. The atomization process led to fairly rounded particles with an average particle size of about 15 µm. The surfaces of the particles observed at higher magnification are shown in the inset of Figure 1a. Marks present at the powder surface, which were created by shrinkage during the solidification process, witness grains having a maximum size of about 10 µm. The nano-sized Mg powder was obtained by an arc-plasma method at NERC-LAF, SJTU (Shanghai, China) by arc evaporation of commercial purity Mg (~99.9%) to generate particle sizes in the sub-micrometer range. In order to preclude these Mg nano-particles from burning in open atmosphere, they were passivated in a mixture of Ar and normal air before collecting them from the evaporation/condensation chamber. The details of this procedure can be found in Ref. [39]. Figure 1b displays representative SEM and tunneling electron microscopy (TEM) images of the condensed powder illustrating the morphological features of the Mg particles. It was estimated from several TEM images that the major particle size distribution was in the range of 50–800 nm with an average particle size below 300 nm, and that the fine powder particles were single crystals with hexagonal symmetry (see the inset of Figure 1b).

Both Mg powder precursors were separately processed by HPT processing at room temperature. A two-step HPT procedure was followed: step 1—a pre-compacted body in disk shape was produced by uniaxial compression of the powder, and step 2—torsional straining by HPT was imposed on the intermediate disk. Both steps were carried out at room temperature. Details can be found in our recent publication [33] where a sketch of the two-step HPT procedure is also given. The powder was first compressed into an intermediate disk with 20 mm in diameter and 3 mm in thickness by uniaxial compression under a pressure of 1.5 GPa and a holding time of 10 min. Subsequent torsional straining was carried out by HPT on the pre-compacted disks under a pressure of 1.2 GPa. This final step was conducted under quasi-constrained conditions [6], where the materials lateral flow is only partially restricted. A total maximum shear strain (γ) of about 42 at the periphery of the disk was achieved by rotating the lower anvil at 0.125 rpm up to 2 revolutions. In the subsequent sections, these HPT-consolidated samples are designated as micro-HPT and nano-HPT product according to their original powder precursors: the atomized micro-sized powder and the condensed nanopowder, respectively.

### 2.2. Characterization

The phases in the initial powders as well as in the HPT-consolidated products were identified by X-ray diffraction (XRD) using a powder X-ray diffraction apparatus (Rigaku, D/max 2550VL/PCX, Tokyo, Japan) using a diffracted beam monochromator and a conventional copper target x-ray tube set to 40 KV and 30 mA. The XRD data were obtained under the following conditions: step size 0.02°, scan speed 10°/min. The JADE software (Jade Software Corp., Christchurch, Nouvelle-Zélande) [40] was utilized to analyze the phase components and the associated crystallographic parameters of the structural elements.

The morphology and the microstructure of the initial powders and their consolidated products were examined by scanning electron microscopy (SEM) using a field emission gun scanning electron microscope (SEM, Zeiss Supra40, Oberkochen, Germany). Mechanical polishing was preliminary carried out on these specimens and was found sufficient to observe the secondary phases, while a subsequent etching with an acetic glycol solution was required to reveal the grain boundaries. The grain orientations and the related microtexture were materialized by using regular electron backscatter diffraction (EBSD) technique for the micro-HPT product, and a SEM-based transmission Kikuchi diffraction (SEM-TKD) facility for the nano-HPT product. For the EBSD preparation, the samples were mechanically polished down to 0.05 μm diamond using a mixture of glycerol and ethanol (Ratio 1 for 3) lubricant and a final rising using pure ethanol. The details of this TKD facility and its acquisition techniques can be found in Ref. [41]. Accordingly, very small size samples were cut from the periphery and from the middle-section of the HPT-disk, then mechanically ground followed by diamond paste cloth polishing. Finally, thin-foils were prepared from the polished samples using a focused ion beam (FIB) technique for the TKD measurements. EBSD maps were treated using the HKL Channel 5 (Oxford Instruments, Abingdon-on-Thames, UK), and the Atom software (Laboratoire d’Etude des Microstructures et de Mécanique des Matériaux, LEM3, Metz, France) [42], while the microtexture based on EBSD measurements was analyzed with the Jtex software (Laboratoire d’Etude des Microstructures et de Mécanique des Matériaux, LEM3, Metz, France) [43].

For hardness measurements, the HPT-disks were cut diagonally, and the cross-sections were polished mechanically with abrasive papers and with cloths using diamond paste to a mirror-like finishing. Vickers microhardness was measured using a Leitz Micro Hardness tester, for a dwell time of 15 s with a load of 50 g. In order to obtain the radial as well as axial distributions of the microhardness, the measurements were conducted at 0.1*R*, 0.3*R*, 0.5*R*, 0.7*R*, and 0.9*R* radial positions in the middle plane and also on some additional cross-sectional planes across the thickness-height (*R* is the radius of the HPT-disk, *R* = 10 mm).

The hydrogen activation characteristics of the two HPT-consolidated products were investigated using a Sievert-type pressure-composition-temperature (PCT) volumetric apparatus (Type PCT-2, Shanghai Institute of Microsystem and Information Technology, Shanghai, China). Prior to hydrogenation, the bulk HPT-products were broken into micrometer size particles (using a ceramic mortar) to fit into the testing vessel. The hydrogen activation was carried out at 400 °C for 8 h under a hydrogen pressure of 3.5 MPa. In order to get insights into the effects of severe plastic deformation (SPD) processing routes on the hydrogen sorption properties of Mg, the initial powder precursors were also tested using identical experimental conditions.

## 3. Results

### 3.1. Structural Characterizations of the Consolidated Products

#### 3.1.1. X-ray Diffraction (XRD)

**Figure 2:**
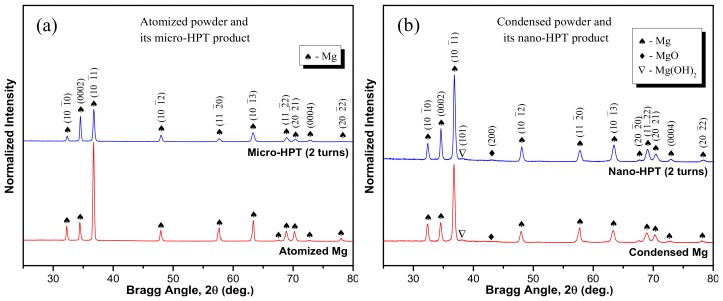
Normalized X-ray diffraction (XRD) patterns recorded on the initial powder precursors and their high-pressure torsion (HPT) products: (**a**) atomized Mg powder and its micro-HPT product; (**b**) condensed Mg powder and its nano-HPT product.

Figure 2 compares the XRD patterns of the initial powder precursors to their HPT-consolidated products. The phase compositions and crystallographic parameters were calculated from the XRD profile refinement using the JADE software [40]; the results are given in Table 1. Besides the major contribution from hexagonal Mg in all XRD profiles, a minor peak from MgO is also detected for the condensed powder and its nano-HPT product (Figure 2b). The amount of oxides in the condensed powder and its nano-HPT product was estimated to be about 5 wt.% (Table 1). In addition, a trace amount of Mg(OH)_2_ was also identified through its (101) reflexion in the condensed powder and its nano-HPT product. This phase along with MgO oxide was formed during the controlled passivation process conducted in the mixture of Ar + normal air. In contrast, the atomized powder and its micro-HPT product did not show such kinds of additional peaks (see Figure 2a), which is due to the lower surface area available for such “contamination.”

The lattice parameters of the Mg phase determined for the initial powders and their HPT-products are listed in Table 1. The results reveal that the lattice constants of the Mg phase in the condensed powder were: a = b = 0.3207 nm and c = 0.5206 nm, while the parameters for its nano-HPT product were a = b = 0.3194 nm and c = 0.5185 nm. The differences in the lattice parameters indicate that there might have been some lattice distortions in the Mg crystal structure during the intense shear straining by HPT. However, these changes in the lattice parameters seem to be less pronounced in case of the micro-HPT product obtained from the less contaminated atomized powder (atomized powder: a = b = 0.3208 nm and c = 0.5209 nm and its micro-HPT product: a = b = 0.3200 nm and c = 0.5192 nm). Furthermore, it is interesting to notice from Table 1 that the HPT consolidation of the powder precursors led to significant differences in their peak intensities. For example, there are considerable differences in the peak intensity ratio of (0002) to (10̅10) planes before and after HPT processing. This ratio substantially increased after HPT treatments of the atomized powder precursor while the increment is not significant for the nano-HPT counterpart. The discrepancy in the peak ratios imply the development of different kinds of textures after shear straining by HPT when different types of powders were used.

#### 3.1.2. SEM Observations

**Figure 3:**
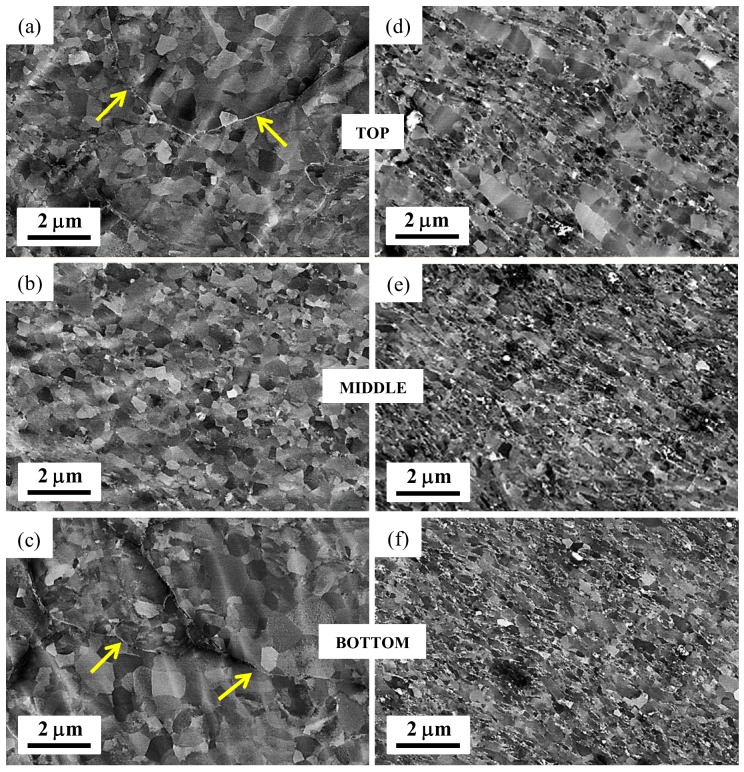
SEM backscattered electron micrographs acquired at the outer-edge and across the through-thickness of the 2 turns HPT disks for (**a**–**c**) the micro-HPT product and (**d**–**f**) the nano-HPT product.

Keeping in mind that the products sintered by HPT consolidation for relatively large thicknesses, such as used in the present study, can be characterized by significant microstructural heterogeneities within their thickness-height [10,33], the SEM study was conducted at the periphery and across the disk-thickness. Typical SEM micrographs obtained under backscattered electron (BSE) imaging conditions are displayed in Figure 3. These images essentially compare the developments of microstructure at the periphery and within the thickness-height for the micro-HPT product, see Figure 3a–c for the nano-HPT product and Figure 3d–f after 2 HPT turns.

In the micro-HPT product consolidated from the atomized powder precursor, the microstructure at the middle-section of the HPT-disk displays a recrystallized equiaxed grain structure, see Figure 3b. This observation is consistent with the results obtained for such a thick sample in the bulk [32], where the shear strain was extremely localized in the middle thickness. This localization of the shear strain induced a dynamic recrystallization process and resulted in an average grain size of 0.5 to 1 µm. In contrast, the top and bottom parts of the micro-HPT disk exhibited fairly heterogeneous microstructures with some deformation-unaffected local domains (Figure 3a,c). This is clearly visible, for example, by the presence of inter-particle oxide layers (as shown by the arrows) that witness the presence of poorly deformed atomized powder particles. Since the presence of the MgO phase was not detected by the XRD measurements for the atomized power (see Figure 2a), it is likely that the oxide content was so small that it did not give rise to any XRD signal under the applied experimental conditions.

**Figure 4:**
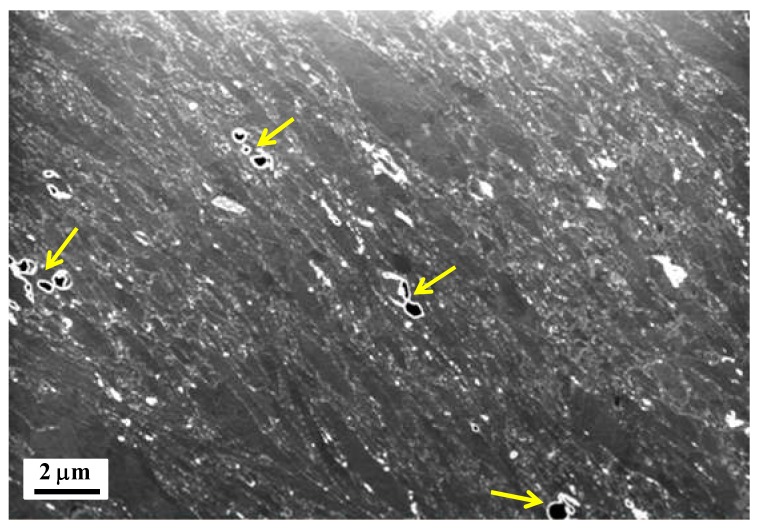
In-situ focused ion beam (FIB) micrograph of the nano-HPT product recorded during the foil preparation for TKD measurements. Arrows indicate the vacancies left after 2 turns HPT consolidation.

In the case of the nano-HPT product, it is readily apparent from the right-side images in Figure 3 that the whole sample was rather more homogeneously deformed, leading to the formation of slightly elongated domains across the disk-thickness (Figure 3d–f). However, the microstructure at the bottom part seems to be less elongated while the width of the Mg domains is thicker at the top; so, a gradient-microstructure developed across the thickness-height. Since the ultrafine powder particles were coated with a thin layer of Mg oxides, it is thereby reasonable to anticipate that these oxide layers were sheared and fragmented into fine particles (appeared as white spots under BSE imaging conditions). Also, it is interesting to notice that most of these oxides were aligned along the grain boundaries that were essentially inclined at about 45°. Figure 4 displays a FIB micrograph obtained from the middle-section of the nano-HPT disks sample prepared for TKD measurements. The microstructural features in the FIB image were consistent with the SEM observations. It is interesting to note that this micrograph clearly shows some residual porosity (as shown by the arrows) even after 2 HPT turns of the nanopowder precursor. It may imply that the densification of the nano-HPT product was not completely achieved.

### 3.2. EBSD and TKD Characterizations

In order to gain some insights into the HPT consolidation deformation behavior of the Mg powder particles, some additional samples were processed at lower level of shear strains (i.e., *N* = 1/2 and 3/4) for both powder systems under identical experimental conditions. Grain orientation mapping for the deformed samples was carefully acquired by using a normal EBSD technique for the micro-HPT product, while a SEM-based TKD technique was used for the nano-HPT products due to their ultra-fine structures. The local EBSD and TKD measurements were carried out at the periphery and on the middle-thickness of the HPT-disk, and the corresponding results are described in the following subsections for the two types of consolidated products.

#### 3.2.1. Micro-HPT Product Consolidated from Atomized Powder

**Figure 5:**
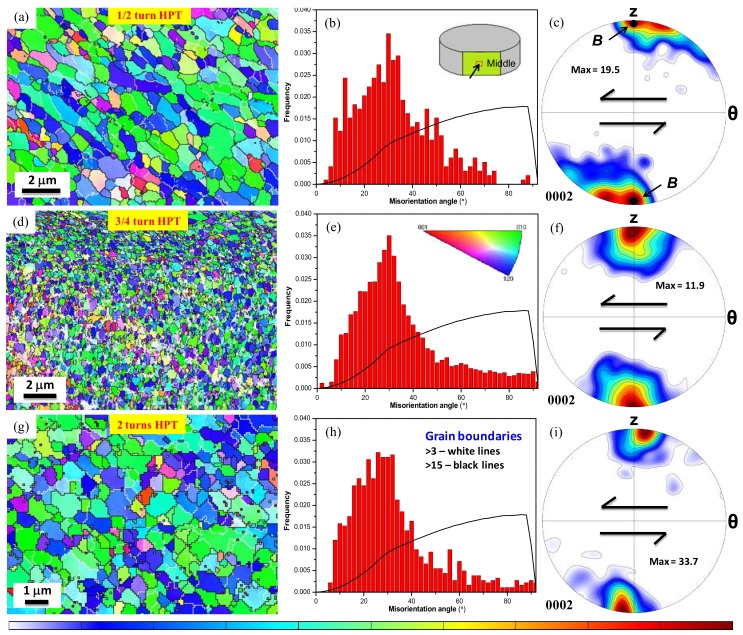
Electron backscatter diffraction (EBSD) orientation images accompanied with grain boundary misorientations and associated microtexture in (0002) pole figure for the 1/2 turn (**a**–**c**), 3/4 turn (**d**–**f**), and 2 turns (**g**–**i**) micro-HPT products acquired at the outer edge and middle-section of the HPT-disk (see the inset of Figure 5b). The Mackenzie distribution of misorientation angles (uncorrelated) is plotted by solid black line. Note the differences in scale bar.

The EBSD orientation maps accompanied with grain misorientation distribution and microtexture for the micro-HPT product deformed up to 1/2, 3/4 and 2 turns are plotted in Figure 5. A schematic diagram of an HPT-disk showing the location and the acquisition direction of the EBSD measurements for all samples is provided in the inset of Figure 5b. The color code in the inset of Figure 5e refers to the crystallographic directions parallel to the normal direction of the sample surface. Furthermore, it should be noted that low-angle grain boundaries (LAGBs, 3–15°) and high-angle grain boundaries (HAGBs, >15°) are denoted by white and black lines, respectively, in all EBSD maps.

The HPT consolidation of atomized powder only up to 1/2 turn has led to the formation of recovered/recrystallized microstructures (see Figure 5a). The grains were elongated at an inclination angle of about 45° from the direction of the imposed shear. Figure 5b displays the grain misorientation angle distribution. A high frequency of medium-angle boundaries can be noticed in the early stages of HPT processing, which is pronounced at around 30°, and can be attributed to the development of a basal fibre texture in Mg materials [44]. Moreover, it appears that the LAGBs are basically limited within the elongated grain domains indicating a close orientation relationship between the neighboring grains, which can be further appreciated from the color gradients within the original grain boundaries. This clearly suggests that, at this stage of deformation, the grain-structure was only partially recovered from its highly deformed state, a phenomenon occurring in materials such as pure Zn, Al, and Mg metal systems deformed at low homologous temperatures [32,36,45]. Figure 5c presents the evolution of the microtexture in (0002) pole figure after 1/2 turn. It is readily apparent that there is a strong *B* fibre, which is the main ideal fibre of hexagonal simple shear textures [46]. It has been observed previously that upon free-end torsion [46], or in HPT processing [47] of pure Mg, the *c*-axis—initially aligned with the shear plane—basically rotates by 90° to become parallel with the torsion axis. In the present study, the *c*-axes of the grains in the powder sample are initially randomly oriented and similarly become significantly aligned with the shear plane normal due to the large shear strain (of about 10 for 1/2 revolution).

Further torsional straining by 3/4 and 2 turns led to recrystallized microstructures accompanied with equiaxed grain-structures (see Figure 5d,g). The elongated shape of the grain-domains after 1/2 HPT turn was transformed into equiaxed grain-structures in the 3/4 turn HPT-product, while the well-defined equiaxed grain-structures in the 2 HPT turns product were inclined at 45° from the shear plane, Figure 5g. These microstructure features are suggesting that the generation of equiaxed microstructures—particularly at the middle-portion—can be associated with the occurrence of complete dynamic recrystallization [32]. The average grain size for these microstructures was in the range of 1 to 1.5 µm for the 3/4 and 0.5 to 1 µm for 2 HPT turns product, respectively. The frequency distribution of grain misorientation shown in Figure 5e and h for the 3/4 and 2 turns infers that most of the grain boundaries were concentrated at around 30°, which also supports the occurrence of a dynamic recovery/recrystallization process. In the case of the 2 HPT turns product, the shear texture component appears tilted from its ideal position. The sense of this tilt is opposite to the direction of the applied shear direction by about 5° (see Figure 5i).

#### 3.2.2. Nano-HPT Product Consolidated from Condensed Powder

**Figure 6:**
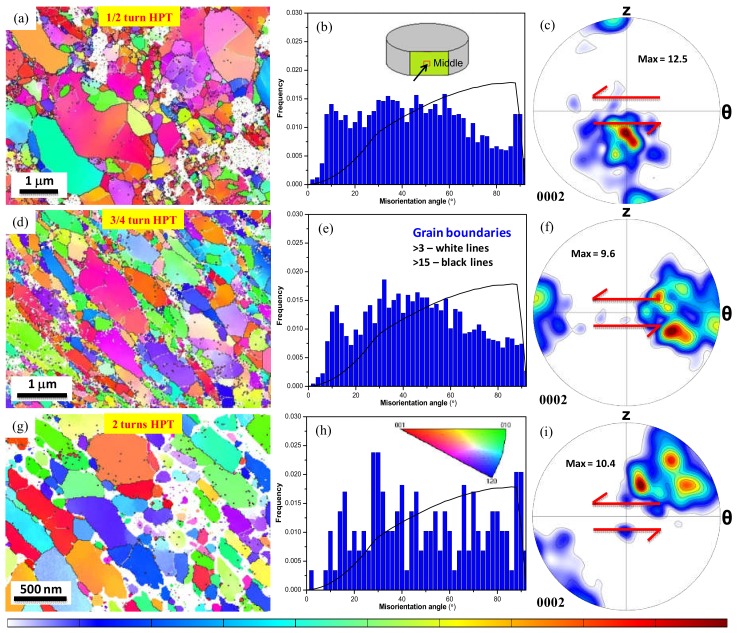
Transmission Kikuchi diffraction (TKD)-based EBSD orientation maps accompanied with grain boundary misorientations and associated microtexture in (0002) pole figure for the 1/2 turn (**a**–**c**), 3/4 turn (**d**–**f**), and 2 turns (**g**–**i**) nano-HPT products, obtained at the outer edge and middle-section of the HPT-disk (see the inset of Figure 6b). Note the differences in scale bar.

The microstructure of the HPT-compacted nanopowder was examined by transmission Kikuchi diffraction (TKD) because of the much smaller grain size. The TKD technique used in our laboratory is unique in the sense that it is applied in a direct on-axis configuration permitting rapid and accurate measurements [41]. At the same time, the maximum size of the examined surface is limited; much smaller than in EBSD. For this reason, the statistical data (disorientation distribution, crystallographic texture) are less representative compared to the EBSD results shown in Figure 5 for the micro-product. Nevertheless, important morphological features and in-grain disorientations can be characterized by these TKD measurements. The evolution of grain orientations and the corresponding microtexture for the nano-HPT product deformed up to 1/2, 3/4, and 2 turns are given in Figure 6.

At the early stages of deformation (i.e., after 1/2 turn), the TKD orientation map in Figure 6a reveals a rather heterogeneous grain-structure consisting of a wide range of grain/particle size distribution. The larger particles likely to be inclined at around 45° to the shear direction while the smaller ones did not respond much to the applied shear strain (i.e., γ = 10 for 1/2 revolution). As mentioned earlier, since the surfaces of ultrafine Mg particles have been originally coated with a thin layer of Mg oxides, these oxide layers were not indexed in the TKD measurements and appeared as non-indexed zones along the particle boundaries. The frequency distribution of grain misorientation shown in Figure 6b clearly indicates mixed type of grain boundaries for this microstructure. As expected from the microstructural heterogeneities, the texture in this sample was not developed as expected from the applied strain (see Figure 6c). The *c*-axes should be positioned at the top and bottom parts of the pole figure in Figure 6c, which is visible. However, large population of grains were very far from these positions.

For the subsequent deformation through 3/4 and 2 turns, a rather homogeneously deformed microstructure was developed in the HPT-products (see Figure 6d,g). In both cases, the grains/particles were significantly elongated with an inclination angle of about 45° from the applied shear. It is readily apparent that some parts of the microstructure seem extremely fragmented to grain sizes less than 100 nm, while the coarser grain-structures were probably generated from initially larger powder particles. Also, the grain/powder particle boundaries, which essentially consisted of oxide layers, were not indexed during the TKD measurements. Since the given TKD map for the 2 HPT turns sample contained a limited number of grains, Figure 6i was constructed from several small TKD maps in order to obtain statistically representative texture using the Jtex software [43].

### 3.3. Microhardness Evolution

**Figure 7:**
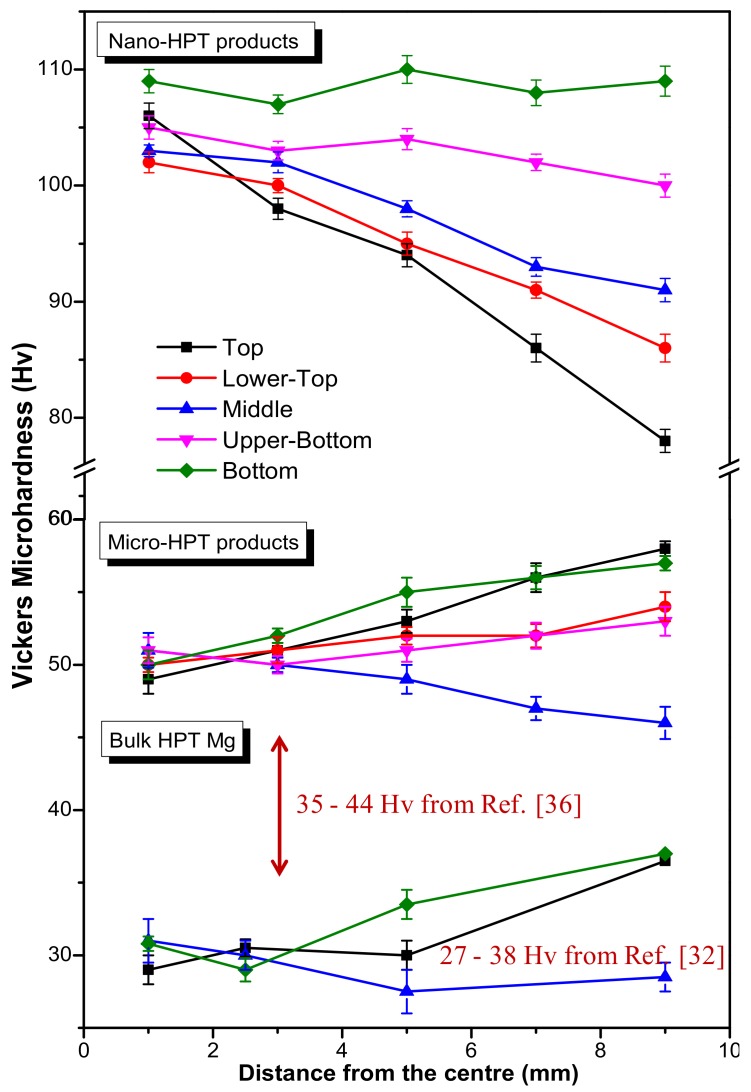
Variations of Vickers microhardness (Hv) along the radial direction as well as across the disk-thickness for the 2 turns nano-HPT and micro-HPT products. The reference hardness values for the HPT deformed bulk Mg samples are also included from Refs. [32,36].

The mechanical strengths of the 2 turns HPT products were characterized by Vickers microhardness across the radial as well as the axial directions; the results are shown in Figure 7. Since it was demonstrated in our recent work [32] that for relatively thick HPT specimens the evolution of microhardness can vary significantly across the through thickness, it was carefully examined on several cross-sectional planes across the thickness height. For comparison, the hardness values obtained for HPT bulk Mg [32,36] are also given in Figure 7.

Because of the presence of finer grains and oxide particles, the hardness of the nano-HPT product is always twice higher than that of the micro-HPT product. It is apparent from Figure 7 for the micro-HPT product that there was a radical drop in the microhardness in the middle section displaying the lowest value at the outer edge of the HPT disk. This decrease in hardness in the mid-thickness is consistent with the observation of microstructural evolutions in the middle-section of the micro-HPT disk (see Figure 3b) in which a recrystallized microstructure developed during the intense shear straining by HPT. Comparatively, there is an increase in hardness values towards the outer-edge for the bottom and top locations. This is due to the fact that while the strain increases towards the periphery of the disk, it does not reach sufficient amount (contrary to what happens at the middle section) to trigger dynamic recrystallization. Such increase in hardness for the HPT-processed sample with increasing strain followed by a hardness drop due to recrystallization was also depicted for pure bulk Mg in Refs. [32,36]. Finally, having an intermediate behavior, the hardness values at the disk center that are associated with comparatively lower strains were almost the same across the disk-thickness.

As recalled by the data given in Figure 7, it is also interesting to notice that the hardness values in our micro-HPT product obtained from atomized powder precursor are always higher than the ones reported on HPT pure Mg for which the precursor was a bulk sample [32,36]. This must be due to the presence of a small fraction of oxide always present at the periphery of the Mg powder particles that provided with some hardening and pinned the grain boundaries to reach finer grain size after deformation. Recently, evaluating the different contributions on strengthening of spark plasma sintered AZ91 Mg alloy, Mondet et al. [20] have reported that the Hall-Petch effect was the major contributor to reach significant improvements in hardness and yield strength.

In the case of the nano-HPT product (see Figure 7), the hardness evolution demonstrates a completely different hardening response than that of its micro-counterpart. Because of the small powder particle size and the presence of oxides, the hardness values were always higher at the disk-center, with an average value of more than 105 Hv for all cross-sectional planes. While the bottom surface exhibited consistently higher hardness values across the radial direction, the other cross-sectional planes showed a monotonic drop in the microhardness values from the disk-centre to the periphery. It is also clearly evident that at the outer-edge of the HPT-disk, the hardness values continuously decreased from the bottom to the top surface. These features are in fact consistent with the microstructural development examined by SEM, where the bottom part revealed a more significant grain refinement than the other parts (see Figure 3d–f).

### 3.4. Hydrogen Activation

**Figure 8:**
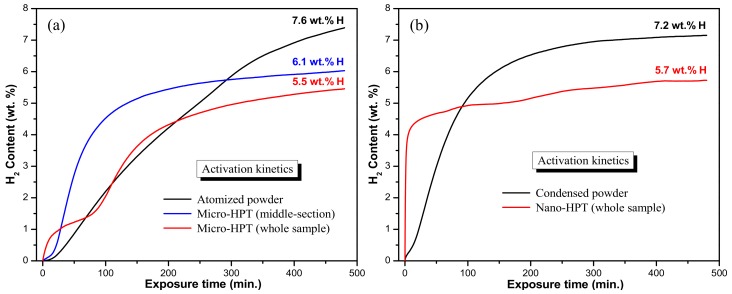
First hydrogenation profiles obtained by dynamic hydrogen absorption of the as-prepared samples at 400 °C for 8 h under a hydrogen pressure of 3.5 MPa: (**a**) atomized powder and its 2 turns micro-HPT product; (**b**) condensed powder and its 2 turns nano-HPT product.

The 2 turns HPT products were analyzed for H-sorption properties during the first hydrogenation. Figure 8 compares the activation characteristics of the two HPT products with that of their initial powder precursors. The hydrogen activation experiments were carried out at 400 °C for 8 h under a hydrogen pressure of 3.5 MPa. Since the micro-HPT disk were characterized by a heterogeneous microstructure across the disk-thickness (see Figure 3a–c), a heterogeneity also materialized by different hardness modifications (see Figure 7), the activation characteristics were tested for the overall sample as well as for the middle section that was recrystallized (Figure 8a). For the nano-HPT product that was more uniformly deformed across the thickness height, the overall sample was tested as a whole (Figure 8b).

Figure 8a shows that the atomized powder particles could store up to the theoretical H-storage capacity of Mg (i.e., 7.6 wt.%) within 8 h (480 min). This is substantially higher than the amount of H stored for the different zones of the micro-HPT product within the same amount of time. While the middle-part of the sample could uptake 6.1 wt.% H, under the identical experimental conditions, the whole sample could reach only 5.5 wt.%. However, the interest of the HPT deformation is that the kinetics of H pick up was improved compared to the loose powder. Within the different bulk micro-HPT parts, the sample obtained from the middle section that was heavily deformed and recrystallized to a fine grain size showed the fastest overall absorption kinetics. Interestingly, the whole sample exhibited a kind of transient behavior with the kinetics slowing down before reaching again a more enhanced absorption rate. Hence, the fine recrystallized microstructures developed in the middle-section enhanced the hydrogenation kinetics compared to the overall performances of the whole sample that consisted of heterogeneous microstructure with a combination of finer recrystallized and coarser deformed grain structures.

For the ultrafine condensed powder and its nano-HPT product (see Figure 8b), the effect of the severe plastic deformation imparted by the HPT processing gave the same trends: an acceleration of the H-storage kinetics but, unfortunately, accompanied with a lower H-storage capacity. While the condensed powder particles took 68 min to reach 4 wt.% of H-uptake, the effect of HPT was that the nano-HPT product required only 5 min for the same 4 wt.% uptake. Concomitantly, the HPT processing has impaired the H-storage capacity of the condensed powder precursor (5.7 wt.% vs. 7.2 wt.% H). 

## 4. Discussion

It was revealed from the present study that the nature of the initial powder precursors had significant impacts on the powder consolidation behavior. The evolutions of the microstructure, the texture and the microhardness for the two HPT-consolidated products were significantly dissimilar in nature. They also showed distinct hydrogen sorption characteristics for the first hydrogenation cycle. These aspects will be discussed hereafter.

### 4.1. Differences in Consolidation Mechanisms, Grain Sizes, and Texture Developments

It has been a great concern for long time that processing of Mg based materials through powder metallurgy routes inherently induces significant difficulties due to the high reactivity of the powder particles towards atmosphere that rapidly generates impervious oxide layer around the powder particles that prevent subsequent sintering [20,22,23]. During HPT processing, huge amount of strain can be introduced into the material assisted by the large applied hydrostatic pressures which can prevent crack formation. As a consequence, the HPT consolidation of powders is mainly governed by particle-particle shearing (rather than diffusion when other thermally assisted sintering processes are used) under which the massive shear straining can fragment the oxide layers and lead to the required bonding, even at low processing temperatures. Concomitantly, while the oxide layer is fractured into nano-sized particles, these oxide particles contributed to the grain size refinement that is known to be one of the major contributors to hardening in Mg alloy [20]. Thus, as illustrated in Figure 3d–f and Figure 4, our nano-HPT composite consolidated from ultrafine condensed and passivated powder particles that contained about 5 wt.% of MgO particles which prevented dynamic recrystallization developed an elongated recovered structure characterized by thin elongated Mg domains pinned by oxide particles. When the HPT process was carried out on the atomized powder that contained a much lower amount of oxide, the material was free to recrystallize if a sufficient amount of plastic deformation was reached. This was the case in the middle portion of the disk, as illustrated in Figure 3b, where a rather refined equiaxed microstructure has formed.

The nature of the powder precursors had significant influences on the development of the microstructure. This is particularly true for the final attainable grain size which depended essentially on the amount of oxide particles present in the deformed products. While a saturation grain size of about 2 to 3 µm is generally obtained for dynamically recrystallized HPT Mg disk deformed from bulk precursors that did not contain oxides [32,36], the HPT consolidation of the atomized Mg powder particles that contained some native oxide at their periphery led to a further refine recrystallized structure with a grain size of 1 µm (see Figure 3b). These differences in grain sizes between the recrystallized products obtained from bulk [32,36] and atomized precursors explain the differences in hardness between these two classes of products, as recalled in Figure 7. As the refinement in grain size is associated with the pinning effect of the oxide particles on the grain boundaries, a finer microstructure developed for the Mg/MgO based nano-HPT product where the grain growth was restricted to elongated Mg domains having an average thickness in the range 0.1 µm (Figure 3f) to 0.5 µm (Figure 3d) depending on the local imparted amount of strain.

As the presence of Mg oxides has affected the mechanisms of deformation and microstructure evolution, it has also affected the texture evolution in the two types of HPT products. A strong shear texture was developed for the micro-HPT product at large shear strains. It clearly implies that the HPT consolidation of the atomized powder particles was mainly governed by simple shear plastic deformation of the powder particles under very high hydrostatic pressure [3,4,5,10]. However, it was noticed, that the location of the *B* fibre was somewhat deviated from its ideal position with increasing shear strain; it was tilted against the shear sense (see Figure 5i). This is consistent with the findings of Bonarski et al. [47] who reported that upon HPT deformation of pure bulk Mg, the *c*-fibre axis deviates from its ideal position and these angular deviation increases while the texture strength decreases by increasing the hydrostatic pressure. According to Skrotzki et al. [48], the occurrence of dynamic recrystallization processes at relatively large shear-strains, as the one taking place in our micro-HPT product, can prevent the rotation of the *c*-axes from reaching the ideal position. Furthermore, the appearance of a large fraction of LAGBs along with the deformed microstructural features after 2 turns suggests that, due to the continuous shear deformation in HPT processing, the grain fragmentation and the associated texture evolution is the consequence of a steady state regime of repeated deformation/recrystallization phenomenon [49,50,51]. In contrast to the micro-HPT product, the texture evolution in the nano-HPT Mg/MgO composite obtained from the condensed and passivated powder particles was more complicated probably due to the influence of the MgO layer that surrounded the Mg core. It was observed from the sequential torsional straining of the condensed powder precursor that the presence of Mg oxide particles somewhat hindered the evolution toward the ideal shear texture in the nano-HPT composite, although a significant densification was achieved through the particle fragmentation followed by adequate bonding between the metallic phases after 2 turns HPT deformation. In spite of the high HPT rotation, the texture depicted in Figure 6 showed many orientations out of the ideal positions. This must imply that many nano-particles did not deform plastically in a proper manner but were probably only rotating against each other under the applied shear.

### 4.2. Effectiveness of Powder Consolidation: Strain Heterogeneities, Associated Microstructure, and Hardness Evolutions

The effectiveness of the powder consolidation was assessed by measuring the relative density of the bulk HPT products using the Archimedes principle; the results are given in Table 2. A relative density of about 92% and 96% was achieved for the micro-HPT and nano-HPT product, respectively, after 2 turns cold consolidation. The lower bulk density for the micro-HPT product can be associated with the relatively poor consolidation at the top and bottom parts of the HPT disk as witnessed from the SEM micrographs (see Figure 3a–c). Indeed, it is well established that products of relatively large thickness (say 3 mm) and deformed by HPT can be characterized by significant microstructure variation due to the development of strain heterogeneities [10,32]. This is reflected here by variations in microstructure and hardness evolutions at different locations of the consolidated product; the hardness being essentially affected by the local grain size and local amount of oxide particles. Thus, the hardness of the nano-HPT product obtained from the condensed and passivated powder exhibited significantly higher microhardness; almost twice higher than the micro-HPT product (nano-HPT: 108 Hv and micro-HPT: 55 Hv). The evolution of the microhardness across the sample volume however demonstrates distinct hardening mechanisms for the two types of HPT products.

The hardening in the micro-HPT product, that contained limited amount of oxide particles, was affected by the occurrence of dynamic recrystallization. Consistently with the results of HPT bulk deformed Mg [32], the maximum amount of plastic strain was obtained in the middle section of the HPT disk where recrystallization (Figure 3b) and an associated softening took place (Figure 7). As the amount of strain increases towards the periphery of the disk, the hardness logically decreases slightly in Figure 7. Comparatively, as confirmed by the presence of some poorly sheared powder particles at the top (Figure 3a) and bottom (Figure 3c) parts of the disk, the amount of strain imparted locally did allow to reach a sufficient amount of plastic deformation to trigger recrystallization. Consequently, the amount of structural defects present in the disk at the bottom and top sections must increase toward the periphery of the disk, and, concomitantly, the hardness increases as well (Figure 7). This hardening followed by a softening behavior at large shear strains is a common feature for pure magnesium [36] as well as for other metal systems having low homologous temperatures that facilitate grain boundary migrations through diffusion processes [45].

The strengthening for the nano-HPT composite is correlated to the presence of the extremely small oxide particles that also pin the grain boundary to prevent recrystallization. Thus, because of the ultra fine size of the condensed powder particles and their core-shell structure coated by a fine layer of oxide, the maximum hardness is always recorded in the center of the disk that was poorly deformed, whatever the height was. In this type of HPT product, the microstructure was less heterogeneous, and the overall disk sustained a fair amount of deformation leading to elongated recovered grains with oxides aligned in the flow direction and pinned their boundaries. However, as for the case depicted in Ref. [10], a strain gradient has developed along the height of the disk from top to bottom. At the bottom of the disk that sustained less strain, the size of the elongated Mg domains remained rather fine, at about 0.1 mm (Figure 3f), and the hardness was rather constant from the center to the periphery of the disk. Comparatively, when going toward the top of the disk, the increasing amount of strain has generated some grain boundary migration that generated recovered elongated Mg domains having larger thicknesses, even up to 0.5 µm (Figure 3d). Consequently, the local average thickness of the elongated Mg domains (Figure 3d–f) and the local hardness developed in the nano-HPT product are directly related to the local amount of strain that was sustained. This is nicely depicted in Figure 7 by the continuous and monotonous decreases in hardness that are visible from the center toward the periphery of the disk, the slope of which increases from the bottom to the top of the disk.

### 4.3. H-Sorption Properties

The first hydrogenation of Mg based material is generally considered to be the most difficult step due to the formation of an impervious oxide layer around the surface of Mg material [37,38]. The activation of the type of powders used in this investigation has already been the subject of previous publications. It was claimed that because of their much finer size and their core-shell structure with the presence of MgO catalysts, the condensed powder particles react much faster than the atomized ones but can store a lower amount of hydrogen. This is confirmed here by comparing the curves of the non-consolidated precursors given in Figure 8a and b for the atomized and condensed powder, respectively.

When properly deformed (i.e., the middle section of the micro-HPT product and the whole nano-HPT one) the HPT-consolidated products always showed faster absorption kinetics in comparison with their original powder precursors. Indeed, the torsional straining of the powder particles introduced numerous structural defects in the Mg matrix (nanostructuring, dislocations, vacancies, etc.) that are known to promote the hydrogen activation [36,37,52,53]. However, this improvement in kinetics is always accompanied by a reduction in the storage capacity. A possible explanation for such behavior lies in the fact that the severe deformation has generated a much higher density of MgH_2_ nucleus at the surface of the components that subsequently led to the formation of a rather continuous and compact layer of hydride at their periphery. The high density of nucleus has improved the initial kinetics but, as was previously demonstrated that hydrogen capacity is drastically influenced by the nucleation rate of the hydride [54,55], must have reduced the capacity because of the low diffusivity of hydrogen through the dense and continuous MgH_2_ layer [56]. When the overall micro-HPT sample that contained also poorly deformed domains was tested, a change in the kinetics was observed during storage so that neither the kinetics nor the amount of H-uptake was improved (red curve in Figure 8a). Concerning the positive effect of HPT, as revealed in a previous study [33], an interesting feature is that it always substantially reduced the hysteresis between the absorption and desorption plateau pressures when these HPT products were subjected to hydrogen uptake/release thermodynamic testing [33,34,35].

## 5. Conclusions

The aim of the present study was to compare the plastic deformation characteristics of 3 mm thick disc obtained by high-pressure torsion (HPT) of two distinct powder precursors: (i) atomized micro-sized Mg (10–70 µm) powder particles containing 2–5 µm grains to consolidate into the so-called micro-HPT disk and (ii) condensed nano-sized Mg (50–800 nm) single grain powder particles that were subsequently passivated to consolidate into the so-called nano-HPT Mg/MgO disk.

The effect of the powder type was examined in terms of (i) microstructure and texture evolution mechanisms, (ii) development of strain heterogeneities and related local mechanical strength (by microhardness), and (iii) H-activation ability by hydrogenation measurements. The following main conclusions are listed hereafter.

1. The nature of the initial powder precursors had significant influences on the microstructure and texture evolutions in the two types of HPT products as well as on the development of local strain heterogeneities.
2. The HPT consolidation of the atomized powder particles led to heterogeneous microstructures across the through thickness: recrystallized equiaxed 1 µm grains at the middle section that sustained the highest strain and rather heterogeneously deformed microstructures at the top and bottom regions of the disk.
3. The HPT consolidation of the condensed powder particles led to a Mg + 5%MgO composite having a rather more homogeneously deformed microstructure consisting of elongated recovered nanometric Mg domains pinned by the nano-sized Mg oxide particles. Some strain heterogeneities have, however, developed from bottom to top of the disk where the average thickness of the elongated recovered grains varied from about 100 to 500 nm.
4. Due to the plastic shear deformation and subsequent dynamic recrystallization, a strong basal texture has developed in the micro-HPT product. Comparatively, the presence of oxides that promoted dynamic recovery and possible powder particle sliding, have hindered the evolution towards the ideal shear texture in the nano-HPT product.
5. The hardness being essentially affected by the local grain size and local amount of oxide particles, the hardness of the nano-HPT product obtained from the fine condensed and passivated powder exhibited twice higher hardness than the micro-HPT product: 108 Hv and 55 Hv, respectively. Also, the hardness values in our micro-HPT product obtained from the atomized powder precursor are always higher than the ones reported on HPT deformed disk of pure Mg for which the precursor was a bulk sample [32,36].
6. The local hardness varied in the consolidated products with the local amount of strain and depended on the mechanism of deformation accommodation. For the micro-HPT product obtained from the atomized powder, the increasing amount of local strain hardened the materials until dynamic recrystallization occurred. Comparatively, for the nano-HPT product obtained from the nano-sized Mg/MgO core-shell powder, the dynamic recovery process led a continuous decrease in local hardness with the amount of strain.
7. After HPT deformation, likely due to an increase in nucleation sites for the hydride formation, the kinetics of the H pick up was significantly improved. For example, the nano-HPT product took only 5 min to reach 4 wt.% H-uptake compared to 68 min for its initial condensed powder precursor. For the heterogeneous micro-HPT product, the fastest kinetics was reported for the central part of the product that was properly deformed and developed a finer recrystallized microstructure. Unfortunately, this faster kinetics was always accompanied with a lower H-storage capacity. For example, in the case of the condensed nano-sized powder, the capacity was reduced from 7.2 wt.% to 5.7 wt.% after HPT.

## Figures and Tables

**Table 1:** Phase composition and crystallographic data for the two types of HPT products obtained from the XRD profile refinement using the JADE software [40].

Samples	Mg (wt.%)	MgO (wt.%)	Mg(OH)_2_ (wt.%)	Lattice Constants (nm)	Intensity Ratio (I_0002_/I_10̅10_)
Atomized Mg	100	-	-	a = b = 0.3208, c = 0.5209	1.24
2 turns micro-HPT	100	-	-	a = b = 0.3200, c = 0.5192	4.11
Condensed Mg	93	5	2	a = b = 0.3207, c = 0.5206	1.08
2 turns nano-HPT	92	5	3	a = b = 0.3194, c = 0.5185	1.73

**Table 2:** Relative density of the HPT products consolidated from the atomized Mg and condensed Mg powder particles.

HPT products	Calculated Density (d_c_, g cm^−3^)	Theoretical Density (d_t_, g cm^−3^)	Relative Density (d_c_/d_t_, %)
2 turns micro-HPT	1.596	1.738	91.8 ± 0.5
2 turns nano-HPT	1.715	1.784 (5 wt.% MgO)	96.1 ± 0.8
